# Oral reovirus therapy modulates gut microbiota to enhance antitumor immunity in colon cancer

**DOI:** 10.1016/j.cpt.2024.12.006

**Published:** 2025-01-03

**Authors:** S. Balachandran, S. Muthamizh, Elangovan Dilipan

**Affiliations:** Department of Physiology, Saveetha Dental College and Hospitals, Saveetha Institute of Medical and Technical Sciences, Saveetha University, Chennai 600077, Tamil Nadu, India

The letter aims to discuss the pivotal findings of the recent study on oncolytic reovirus (RC402) focusing on its administration route and how this route enhances immunotherapeutic efficacy against advanced cancers. The insights gained from this study regarding oral reovirus treatment improve our understanding of oncolytic virotherapy and propose a feasible strategy for enhancing the efficacy of cancer immunotherapy. The research demonstrates that oral administration of RC402 can effectively induce antitumor immunity without significant toxicities. This finding is particularly important, as conventional treatments often entail adverse effects that negatively impact a patient's quality of life. The study findings indicate that the orally administered reovirus interacts intricately with the host immune system, particularly within the Peyer's patches of the terminal ileum. This interaction leads to an increase in immunoglobulin A (IgA^+^)-secreting cells in the lamina propria via mucosal addressin cell adhesion molecule-1 (MAdCAM-1^+^) blood vessels.^1^ This mechanism highlights a novel pathway that can be used to leverage the gut-associated lymphoid tissue to initiate systemic immune responses against tumors.

Furthermore, the research demonstrates that oral reovirus administration enhances antigen presentation, promotes type I and II interferon production, and activates T cells in distant tumors. Notably, this treatment approach does not rely on the direct infection of tumor cells beyond the gastrointestinal tract; immune activation appears to be mediated through complex interactions between the reovirus and the gut microbiome and also involves basic leucine zipper ATF-like transcription factor 3 (Batf3+) dendritic cells, type I interferons, and CD8+ T cells.^2^ This phenomenon is in stark contrast with the one observed upon intratumoral injection of reovirus, which directly targets tumor cells but may not exploit the systemic immune modulation that is observed with oral administration. Additionally, the study highlights the enhanced effectiveness of oral reovirus treatment when combined with immune checkpoint inhibitors, such as anti-programmed death-1 antibody (anti-PD-1[L1]) and/or anti-cytotoxic T-lymphocyte-associated protein 4 antibody (anti-CTLA-4).^3^ This combination therapy resulted in the complete regression of colon tumors and established protective immune memory, presenting a compelling case for integrating oral virotherapy into existing treatment paradigms.

Given the rising global incidence of cancer, particularly in regions such as India where gastrointestinal malignancies are prevalent, the implications of this research are profound. Patients often encounter challenges in accessing advanced therapies that require invasive procedures. The oral administration route presents a more accessible alternative, potentially increasing patient compliance and expanding treatment options for those with advanced cancer.

In India, where socioeconomic barriers often hinder access to cancer treatment, the development of non-invasive, effective immunotherapeutic strategies, such as oral reovirus virotherapy, could have a transformative impact on cancer care. As we strive to improve therapeutic outcomes, incorporating strategies that utilize the gut microbiome and systemic immune responses may boost efficacy and reduce treatment-related toxicities.^4^

In conclusion, the findings of this preclinical study lay a foundation for future research into oral oncolytic virotherapy as a promising viable immunotherapeutic strategy. We encourage the scientific community to delve deeper into this promising approach, as it holds great potential in transforming cancer treatment and improving patient outcomes worldwide, particularly in high-risk populations.

## Authors contributions

Balachandran S: writing - original draft, data curation, and conceptualization; Muthamizh S and Dilipan E: writing - review and editing, methodology, and supervision. All the authors have read and approved the final version of the manuscript.

## Ethics statement

None.

## Declaration of generative AI and AI-assisted technologies in the writing process

The authors declare that generative artificial intelligence (AI) and AI assisted technologies were not used in the writing process or any other process during the preparation of this manuscript.

## Funding

None.

## Data availability statement

The datasets used in the current study are available from the corresponding author on reasonable request.

## Conflict of interest

The authors declare that they have no known competing financial interests or personal relationships that could have appeared to influence the work reported in this paper.
